# Varenicline-induced sucidal behavior: Case report and literature review

**DOI:** 10.1192/j.eurpsy.2022.1844

**Published:** 2022-09-01

**Authors:** T. Gutierrez Higueras, F. Calera Cortés, S. Sainz De La Cuesta Alonso, S. Vicent Forés, B. Hernández Gajate, R.M. Fiestas Velasco

**Affiliations:** 1 Reina Sofia University Hospital, Psychiatry, Córdoba, Spain; 2 Hospital Reina Sofía Córdoba, Psychiatry, Cordoba, Spain; 3 Hospital Reina Sofía Córdoba, Psychiatry, Córdoba, Spain

**Keywords:** Varenicline, suicidal behavior, smoking cessation, symptoms

## Abstract

**Introduction:**

Nowdays there are different strategies for the treatment of smoking cessation. The treatment include drugs such as varenicline, which acts as a high-affinity partial agonist for the alpha-4 beta-2 nicotinic acetylcholine receptor subtype (nACh). We report a case of a suicidal behaviour in a 39 year-old woman with no previous history of mental illness, who was brought to the emergency department after intentional intoxication with benzodiazepines. The patient was on 10th day of treatment with varenicline.

**Objectives:**

To present a case of sucidal behavior that developed in a 39 year-old woman after starting varenicline. Review of literature and total number of cases reported in the european database of suspected adverse drug reactions (EudraVigilance).

**Methods:**

We carried out a literature review in Pubmed electing those articles focused on mental disorders in those patients that have been taking varenicline. Review number of cases suicidal behavior reported by the European database of suspected adverse drug reactions.

**Results:**

A 39-year-old female was brought to the emergency department after voluntary ingestion of Lorazepam 1mg (40 tablets) in a sucide attempt. The family reported the starting of thoughts of suicide after 1 week of treatment. No previous history of mental disorders. The patient reported low mood and drowsiness in the last 5 days not linked to any cause. After 5 days of discontinuation these mood symptoms and sucidal behavior remited.

**Conclusions:**

Varenicline is associated with different neuropsychiatric sypmtoms. In patients with or without history of mental disorders we should warn about the symptoms for discontinuation of the treatment.

**Disclosure:**

No significant relationships.

